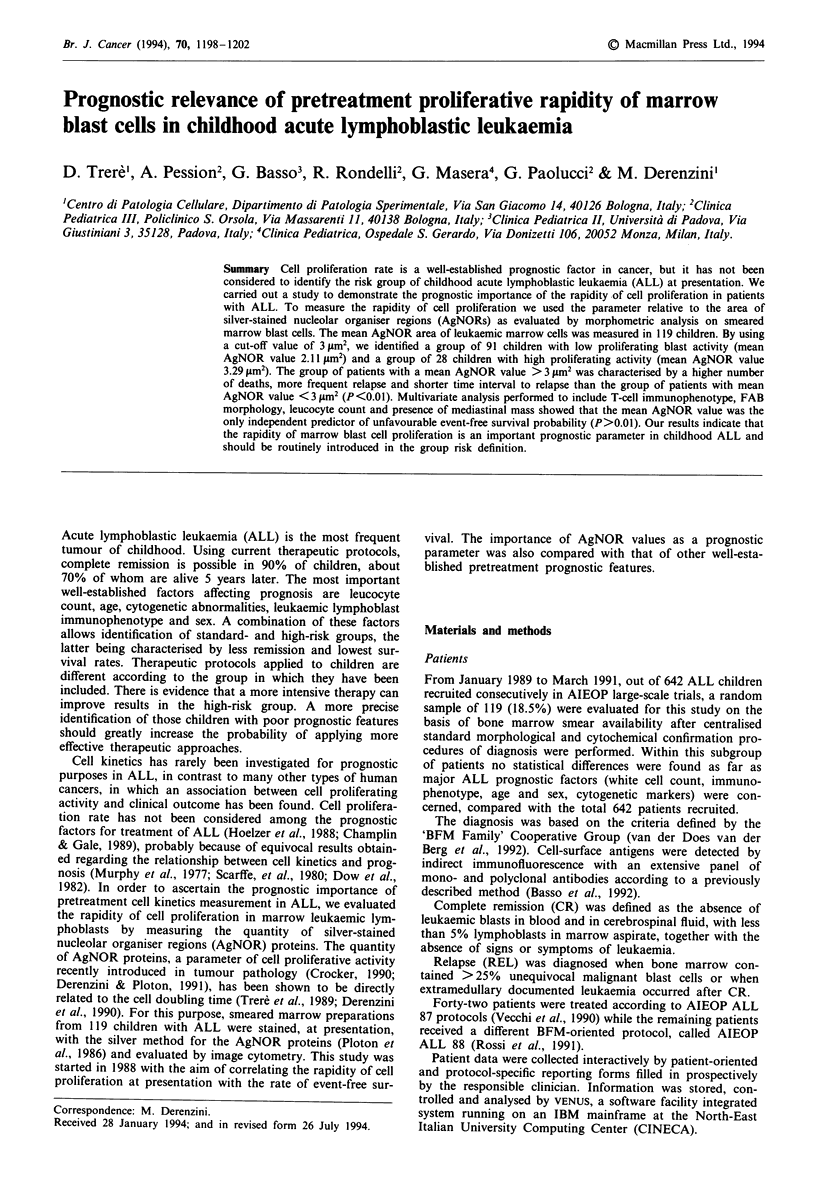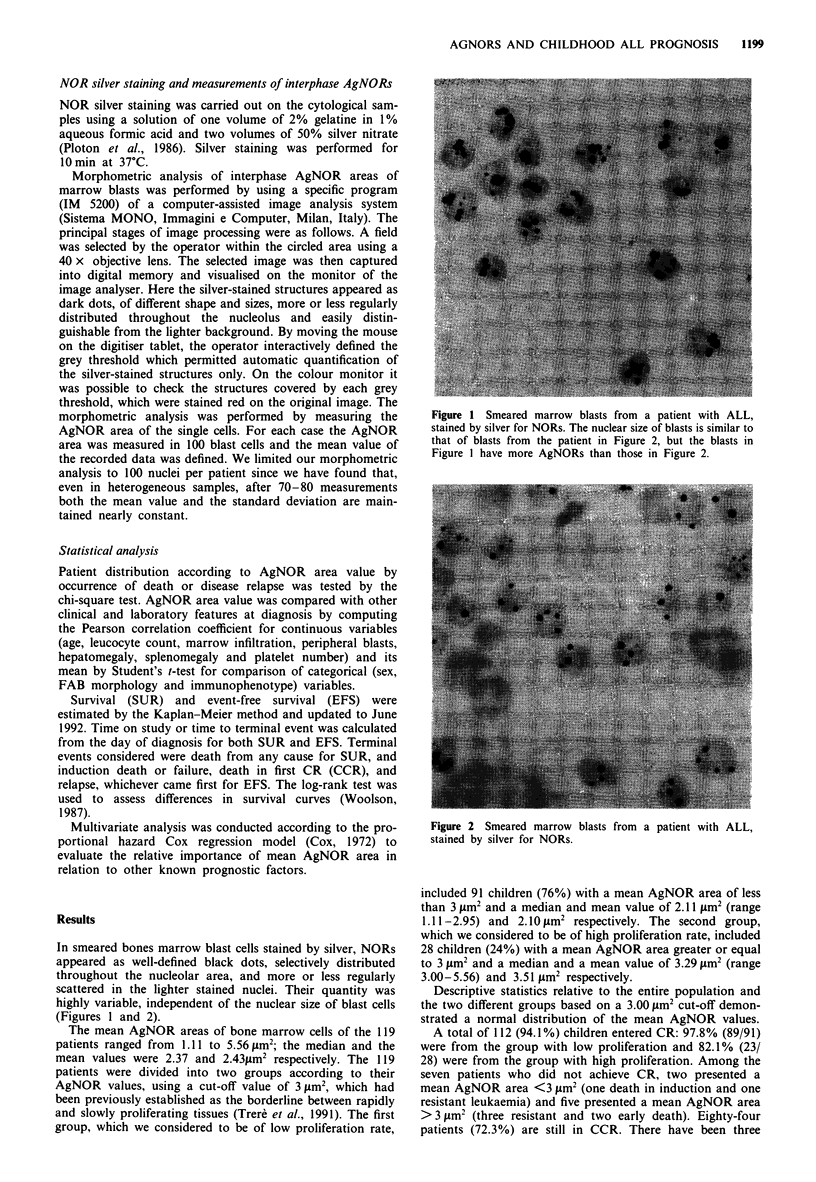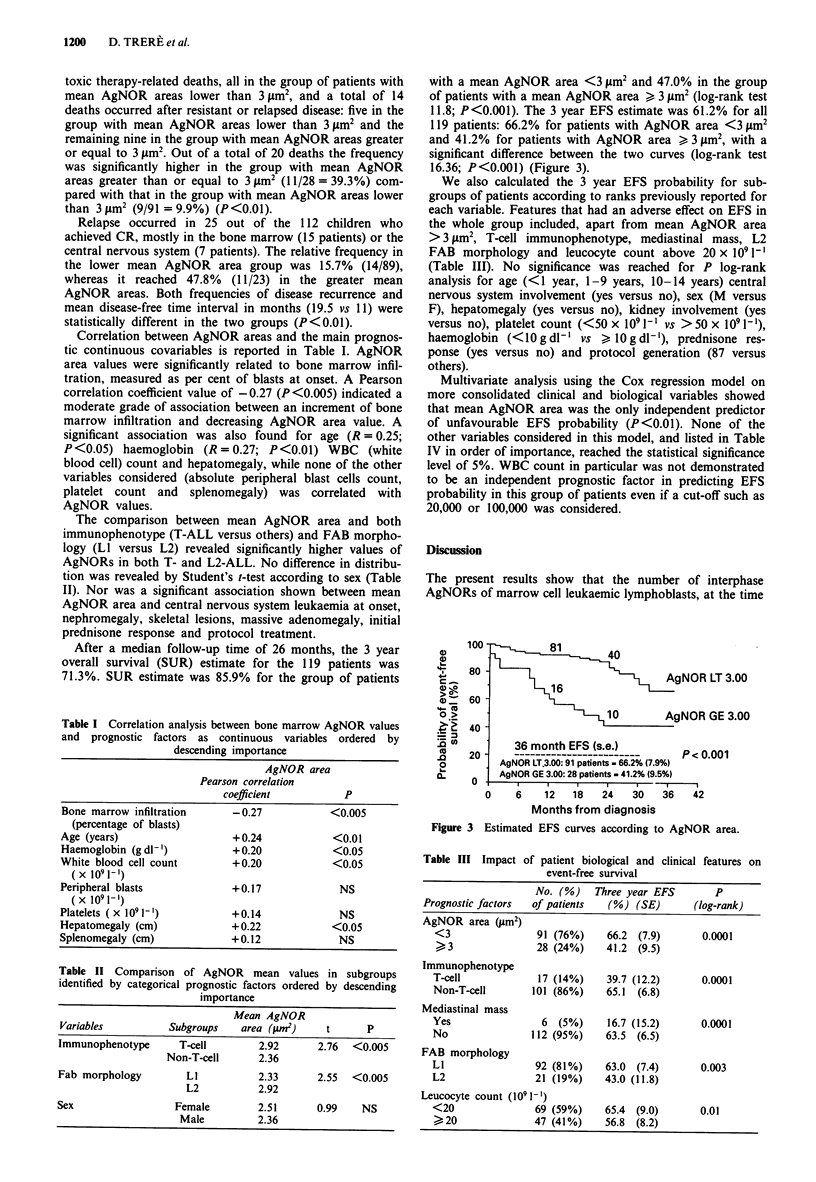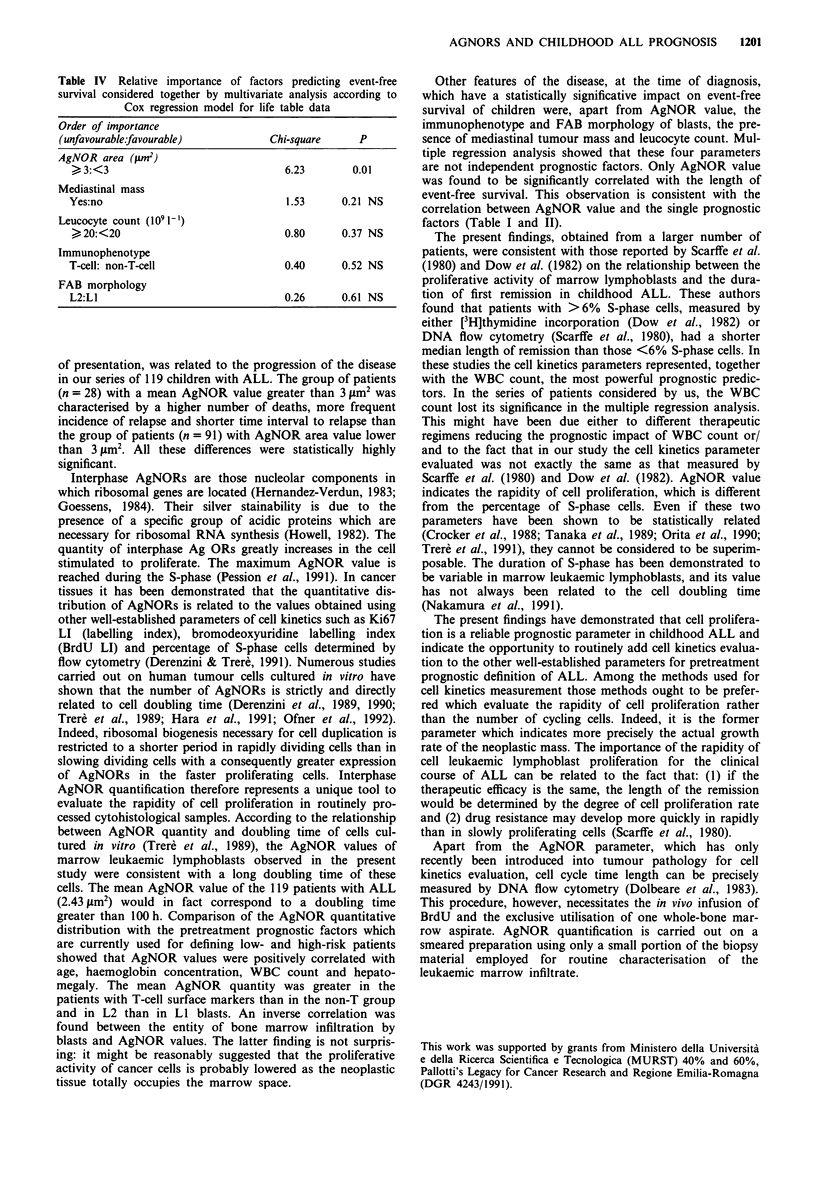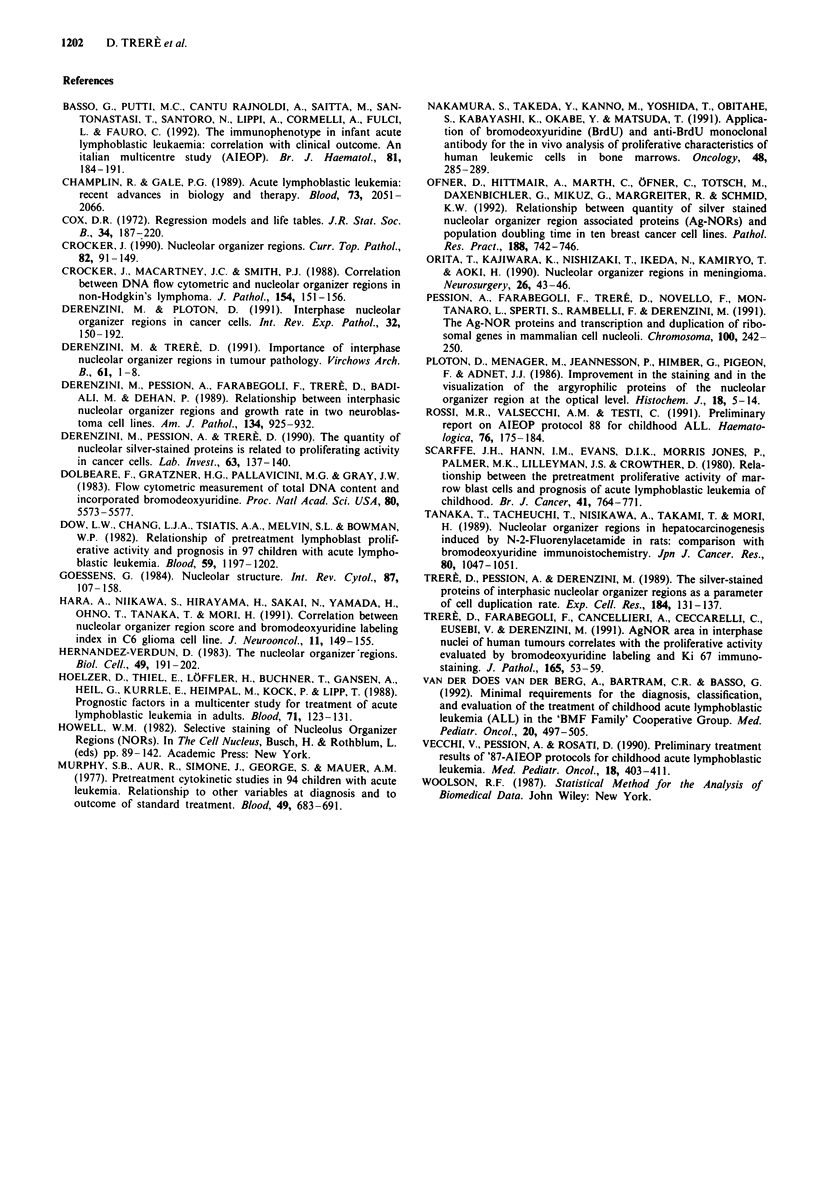# Prognostic relevance of pretreatment proliferative rapidity of marrow blast cells in childhood acute lymphoblastic leukaemia.

**DOI:** 10.1038/bjc.1994.473

**Published:** 1994-12

**Authors:** D. Trerè, A. Pession, G. Basso, R. Rondelli, G. Masera, G. Paolucci, M. Derenzini

**Affiliations:** Centro di Patologia Cellulare, Dipartimento di Patologia Sperimentale, Bologna, Italy.

## Abstract

**Images:**


Br. J. Cancer (1994), 70, 1198-1202 © Macmillan Press Ltd., 1994

### Prognostic relevance of pretreatment proliferative rapidity of marrow blast cells in childhood acute lymphoblastic leukaemia

D. Trerè[1], A. Pession[2], G. Basso[3], R. Rondelli[2], G. Masera[4], G. Paolucci[2] & M. Derenzini[1]

[1]*Centro di Patologia Cellulare, Dipartimento di Patologia Sperimentale, Via San Giacomo 14, 40126 Bologna, Italy;* [2]*Clinica Pediatrica III, Policlinico S. Orsola, Via Massarenti 11, 40138 Bologna, Italy;* [3]*Clinica Pediatrica II, Università di Padova, Via Giustiniani 3, 35128, Padova, Italy;* [4]*Clinica Pediatrica, Ospedale S. Gerardo, Via Donizetti 106, 20052 Monza, Milan, Italy.*

**Summary** Cell proliferation rate is a well-established prognostic factor in cancer, but it has not been considered to identify the risk group of childhood acute lymphoblastic leukaemia (ALL) at presentation. We carried out a study to demonstrate the prognostic importance of the rapidity of cell proliferation in patients with ALL. To measure the rapidity of cell proliferation we used the parameter relative to the area of silver-stained nucleolar organiser regions (AgNORs) as evaluated by morphometric analysis on smeared marrow blast cells. The mean AgNOR area of leukaemic marrow cells was measured in 119 children. By using a cut-off value of $3\,\mu m^2$, we identified a group of 91 children with low proliferating blast activity (mean AgNOR value $2.11\,\mu m^2$) and a group of 28 children with high proliferating activity (mean AgNOR value $3.29\,\mu m^2$). The group of patients with a mean AgNOR value $>3\,\mu m^2$ was characterised by a higher number of deaths, more frequent relapse and shorter time interval to relapse than the group of patients with mean AgNOR value $<3\,\mu m^2$ ($P<0.01$). Multivariate analysis performed to include T-cell immunophenotype, FAB morphology, leucocyte count and presence of mediastinal mass showed that the mean AgNOR value was the only independent predictor of unfavourable event-free survival probability ($P>0.01$). Our results indicate that the rapidity of marrow blast cell proliferation is an important prognostic parameter in childhood ALL and should be routinely introduced in the group risk definition.

Acute lymphoblastic leukaemia (ALL) is the most frequent tumour of childhood. Using current therapeutic protocols, complete remission is possible in 90% of children, about 70% of whom are alive 5 years later. The most important well-established factors affecting prognosis are leucocyte count, age, cytogenetic abnormalities, leukaemic lymphoblast immunophenotype and sex. A combination of these factors allows identification of standard- and high-risk groups, the latter being characterised by less remission and lowest survival rates. Therapeutic protocols applied to children are different according to the group in which they have been included. There is evidence that a more intensive therapy can improve results in the high-risk group. A more precise identification of those children with poor prognostic features should greatly increase the probability of applying more effective therapeutic approaches.

Cell kinetics has rarely been investigated for prognostic purposes in ALL, in contrast to many other types of human cancers, in which an association between cell proliferating activity and clinical outcome has been found. Cell proliferation rate has not been considered among the prognostic factors for treatment of ALL (Hoelzer *et al.*, 1988; Champlin & Gale, 1989), probably because of equivocal results obtained regarding the relationship between cell kinetics and prognosis (Murphy *et al.*, 1977; Scarffe, *et al.*, 1980; Dow *et al.*, 1982). In order to ascertain the prognostic importance of pretreatment cell kinetics measurement in ALL, we evaluated the rapidity of cell proliferation in marrow leukaemic lymphoblasts by measuring the quantity of silver-stained nucleolar organiser regions (AgNOR) proteins. The quantity of AgNOR proteins, a parameter of cell proliferative activity recently introduced in tumour pathology (Crocker, 1990; Derenzini & Ploton, 1991), has been shown to be directly related to the cell doubling time (Trerè *et al.*, 1989; Derenzini *et al.*, 1990). For this purpose, smeared marrow preparations from 119 children with ALL were stained, at presentation, with the silver method for the AgNOR proteins (Ploton *et al.*, 1986) and evaluated by image cytometry. This study was started in 1988 with the aim of correlating the rapidity of cell proliferation at presentation with the rate of event-free survival. The importance of AgNOR values as a prognostic parameter was also compared with that of other well-established pretreatment prognostic features.

Correspondence: M. Derenzini.
Received 28 January 1994; and in revised form 26 July 1994.

### Materials and methods

### *Patients*

From January 1989 to March 1991, out of 642 ALL children recruited consecutively in AIEOP large-scale trials, a random sample of 119 (18.5%) were evaluated for this study on the basis of bone marrow smear availability after centralised standard morphological and cytochemical confirmation procedures of diagnosis were performed. Within this subgroup of patients no statistical differences were found as far as major ALL prognostic factors (white cell count, immunophenotype, age and sex, cytogenetic markers) were concerned, compared with the total 642 patients recruited.

The diagnosis was based on the criteria defined by the 'BFM Family' Cooperative Group (van der Does van der Berg *et al.*, 1992). Cell-surface antigens were detected by indirect immunofluorescence with an extensive panel of mono- and polyclonal antibodies according to a previously described method (Basso *et al.*, 1992).

Complete remission (CR) was defined as the absence of leukaemic blasts in blood and in cerebrospinal fluid, with less than 5% lymphoblasts in marrow aspirate, together with the absence of signs or symptoms of leukaemia.

Relapse (REL) was diagnosed when bone marrow contained >25% unequivocal malignant blast cells or when extramedullary documented leukaemia occurred after CR.

Forty-two patients were treated according to AIEOP ALL 87 protocols (Vecchi *et al.*, 1990) while the remaining patients received a different BFM-oriented protocol, called AIEOP ALL 88 (Rossi *et al.*, 1991).

Patient data were collected interactively by patient-oriented and protocol-specific reporting forms filled in prospectively by the responsible clinician. Information was stored, controlled and analysed by VENUS, a software facility integrated system running on an IBM mainframe at the North-East Italian University Computing Center (CINECA).

### *NOR silver staining and measurements of interphase AgNORs*

NOR silver staining was carried out on the cytological samples using a solution of one volume of 2% gelatine in 1% aqueous formic acid and two volumes of 50% silver nitrate (Ploton *et al.*, 1986). Silver staining was performed for 10 min at 37°C.

Morphometric analysis of interphase AgNOR areas of marrow blasts was performed by using a specific program (IM 5200) of a computer-assisted image analysis system (Sistema MONO, Immagini e Computer, Milan, Italy). The principal stages of image processing were as follows. A field was selected by the operator within the circled area using a 40 × objective lens. The selected image was then captured into digital memory and visualised on the monitor of the image analyser. Here the silver-stained structures appeared as dark dots, of different shape and sizes, more or less regularly distributed throughout the nucleolus and easily distinguishable from the lighter background. By moving the mouse on the digitiser tablet, the operator interactively defined the grey threshold which permitted automatic quantification of the silver-stained structures only. On the colour monitor it was possible to check the structures covered by each grey threshold, which were stained red on the original image. The morphometric analysis was performed by measuring the AgNOR area of the single cells. For each case the AgNOR area was measured in 100 blast cells and the mean value of the recorded data was defined. We limited our morphometric analysis to 100 nuclei per patient since we have found that, even in heterogeneous samples, after 70–80 measurements both the mean value and the standard deviation are maintained nearly constant.

### *Statistical analysis*

Patient distribution according to AgNOR area value by occurrence of death or disease relapse was tested by the chi-square test. AgNOR area value was compared with other clinical and laboratory features at diagnosis by computing the Pearson correlation coefficient for continuous variables (age, leucocyte count, marrow infiltration, peripheral blasts, hepatomegaly, splenomegaly and platelet number) and its mean by Student's *t*-test for comparison of categorical (sex, FAB morphology and immunophenotype) variables.

Survival (SUR) and event-free survival (EFS) were estimated by the Kaplan–Meier method and updated to June 1992. Time on study or time to terminal event was calculated from the day of diagnosis for both SUR and EFS. Terminal events considered were death from any cause for SUR, and induction death or failure, death in first CR (CCR), and relapse, whichever came first for EFS. The log-rank test was used to assess differences in survival curves (Woolson, 1987).

Multivariate analysis was conducted according to the proportional hazard Cox regression model (Cox, 1972) to evaluate the relative importance of mean AgNOR area in relation to other known prognostic factors.

**Figure 1** Smeared marrow blasts from a patient with ALL, stained by silver for NORs. The nuclear size of blasts is similar to that of blasts from the patient in Figure 2, but the blasts in Figure 1 have more AgNORs than those in Figure 2.

**Figure 2** Smeared marrow blasts from a patient with ALL, stained by silver for NORs.

### Results

In smeared bones marrow blast cells stained by silver, NORs appeared as well-defined black dots, selectively distributed throughout the nucleolar area, and more or less regularly scattered in the lighter stained nuclei. Their quantity was highly variable, independent of the nuclear size of blast cells (Figures 1 and 2).

The mean AgNOR areas of bone marrow cells of the 119 patients ranged from 1.11 to 5.56 $\mu m^2$; the median and the mean values were 2.37 and 2.43 $\mu m^2$ respectively. The 119 patients were divided into two groups according to their AgNOR values, using a cut-off value of 3 $\mu m^2$, which had been previously established as the borderline between rapidly and slowly proliferating tissues (Trerè *et al.*, 1991). The first group, which we considered to be of low proliferation rate, included 91 children (76%) with a mean AgNOR area of less than 3 $\mu m^2$ and a median and mean value of 2.11 $\mu m^2$ (range 1.11–2.95) and 2.10 $\mu m^2$ respectively. The second group, which we considered to be of high proliferation rate, included 28 children (24%) with a mean AgNOR area greater or equal to 3 $\mu m^2$ and a median and a mean value of 3.29 $\mu m^2$ (range 3.00–5.56) and 3.51 $\mu m^2$ respectively.

Descriptive statistics relative to the entire population and the two different groups based on a 3.00 $\mu m^2$ cut-off demonstrated a normal distribution of the mean AgNOR values.

A total of 112 (94.1%) children entered CR: 97.8% (89/91) were from the group with low proliferation and 82.1% (23/28) were from the group with high proliferation. Among the seven patients who did not achieve CR, two presented a mean AgNOR area $<3\ \mu m^2$ (one death in induction and one resistant leukaemia) and five presented a mean AgNOR area $>3\ \mu m^2$ (three resistant and two early death). Eighty-four patients (72.3%) are still in CCR. There have been three toxic therapy-related deaths, all in the group of patients with mean AgNOR areas lower than 3 μm², and a total of 14 deaths occurred after resistant or relapsed disease: five in the group with mean AgNOR areas lower than 3 μm² and the remaining nine in the group with mean AgNOR areas greater or equal to 3 μm². Out of a total of 20 deaths the frequency was significantly higher in the group with mean AgNOR areas greater than or equal to 3 μm² (11/28 = 39.3%) compared with that in the group with mean AgNOR areas lower than 3 μm² (9/91 = 9.9%) ($P<0.01$).

Relapse occurred in 25 out of the 112 children who achieved CR, mostly in the bone marrow (15 patients) or the central nervous system (7 patients). The relative frequency in the lower mean AgNOR area group was 15.7% (14/89), whereas it reached 47.8% (11/23) in the greater mean AgNOR areas. Both frequencies of disease recurrence and mean disease-free time interval in months (19.5 *vs* 11) were statistically different in the two groups ($P<0.01$).

Correlation between AgNOR areas and the main prognostic continuous covariables is reported in Table I. AgNOR area values were significantly related to bone marrow infiltration, measured as per cent of blasts at onset. A Pearson correlation coefficient value of −0.27 ($P<0.005$) indicated a moderate grade of association between an increment of bone marrow infiltration and decreasing AgNOR area value. A significant association was also found for age ($R = 0.25$; $P<0.05$) haemoglobin ($R = 0.27$; $P<0.01$) WBC (white blood cell) count and hepatomegaly, while none of the other variables considered (absolute peripheral blast cells count, platelet count and splenomegaly) was correlated with AgNOR values.

The comparison between mean AgNOR area and both immunophenotype (T-ALL versus others) and FAB morphology (L1 versus L2) revealed significantly higher values of AgNORs in both T- and L2-ALL. No difference in distribution was revealed by Student's *t*-test according to sex (Table II). Nor was a significant association shown between mean AgNOR area and central nervous system leukaemia at onset, nephromegaly, skeletal lesions, massive adenomegaly, initial prednisone response and protocol treatment.

After a median follow-up time of 26 months, the 3 year overall survival (SUR) estimate for the 119 patients was 71.3%. SUR estimate was 85.9% for the group of patients with a mean AgNOR area <3 μm² and 47.0% in the group of patients with a mean AgNOR area ⩾ 3 μm² (log-rank test 11.8; $P<0.001$). The 3 year EFS estimate was 61.2% for all 119 patients: 66.2% for patients with AgNOR area <3 μm² and 41.2% for patients with AgNOR area ⩾ 3 μm², with a significant difference between the two curves (log-rank test 16.36; $P<0.001$) (Figure 3).

We also calculated the 3 year EFS probability for subgroups of patients according to ranks previously reported for each variable. Features that had an adverse effect on EFS in the whole group included, apart from mean AgNOR area > 3 μm², T-cell immunophenotype, mediastinal mass, L2 FAB morphology and leucocyte count above $20 \times 10^9 l^{-1}$ (Table III). No significance was reached for *P* log-rank analysis for age (<1 year, 1–9 years, 10–14 years) central nervous system involvement (yes versus no), sex (M versus F), hepatomegaly (yes versus no), kidney involvement (yes versus no), platelet count ($<50 \times 10^9 l^{-1}$ *vs* $>50 \times 10^9 l^{-1}$), haemoglobin ($<10 g dl^{-1}$ *vs* $\geq 10 g dl^{-1}$), prednisone response (yes versus no) and protocol generation (87 versus others).

Multivariate analysis using the Cox regression model on more consolidated clinical and biological variables showed that mean AgNOR area was the only independent predictor of unfavourable EFS probability ($P<0.01$). None of the other variables considered in this model, and listed in Table IV in order of importance, reached the statistical significance level of 5%. WBC count in particular was not demonstrated to be an independent prognostic factor in predicting EFS probability in this group of patients even if a cut-off such as 20,000 or 100,000 was considered.

**Table I** Correlation analysis between bone marrow AgNOR values and prognostic factors as continuous variables ordered by descending importance

| | *AgNOR area* Pearson correlation coefficient | *P* |
|---|---|---|
| Bone marrow infiltration (percentage of blasts) | −0.27 | <0.005 |
| Age (years) | +0.24 | <0.01 |
| Haemoglobin ($g dl^{-1}$) | +0.20 | <0.05 |
| White blood cell count ($\times 10^9 l^{-1}$) | +0.20 | <0.05 |
| Peripheral blasts ($\times 10^9 l^{-1}$) | +0.17 | NS |
| Platelets ($\times 10^9 l^{-1}$) | +0.14 | NS |
| Hepatomegaly (cm) | +0.22 | <0.05 |
| Splenomegaly (cm) | +0.12 | NS |

**Table II** Comparison of AgNOR mean values in subgroups identified by categorical prognostic factors ordered by descending importance

| *Variables* | *Subgroups* | *Mean AgNOR area (μm²)* | t | P |
|---|---|---|---|---|
| Immunophenotype | T-cell | 2.92 | 2.76 | <0.005 |
| | Non-T-cell | 2.36 | | |
| Fab morphology | L1 | 2.33 | 2.55 | <0.005 |
| | L2 | 2.92 | | |
| Sex | Female | 2.51 | 0.99 | NS |
| | Male | 2.36 | | |

### Discussion

The present results show that the number of interphase AgNORs of marrow cell leukaemic lymphoblasts, at the time

**Figure 3** Estimated EFS curves according to AgNOR area.

**Table III** Impact of patient biological and clinical features on event-free survival

| *Prognostic factors* | *No. (%) of patients* | *Three year EFS (%) (SE)* | *P (log-rank)* |
|---|---|---|---|
| AgNOR area (μm²) | | | |
| <3 | 91 (76%) | 66.2 (7.9) | 0.0001 |
| ⩾3 | 28 (24%) | 41.2 (9.5) | |
| Immunophenotype | | | |
| T-cell | 17 (14%) | 39.7 (12.2) | 0.0001 |
| Non-T-cell | 101 (86%) | 65.1 (6.8) | |
| Mediastinal mass | | | |
| Yes | 6 (5%) | 16.7 (15.2) | 0.0001 |
| No | 112 (95%) | 63.5 (6.5) | |
| FAB morphology | | | |
| L1 | 92 (81%) | 63.0 (7.4) | 0.003 |
| L2 | 21 (19%) | 43.0 (11.8) | |
| Leucocyte count ($10^9 l^{-1}$) | | | |
| <20 | 69 (59%) | 65.4 (9.0) | 0.01 |
| ⩾20 | 47 (41%) | 56.8 (8.2) | |

**Table IV** Relative importance of factors predicting event-free survival considered together by multivariate analysis according to Cox regression model for life table data

| *Order of importance (unfavourable:favourable)* | *Chi-square* | *P* |
|---|---|---|
| *AgNOR area* ($\mu m^2$) | | |
| $\geqslant 3:<3$ | 6.23 | 0.01 |
| Mediastinal mass | | |
| Yes:no | 1.53 | 0.21 NS |
| Leucocyte count ($10^9 l^{-1}$) | | |
| $\geqslant 20:<20$ | 0.80 | 0.37 NS |
| Immunophenotype | | |
| T-cell: non-T-cell | 0.40 | 0.52 NS |
| FAB morphology | | |
| L2:L1 | 0.26 | 0.61 NS |

of presentation, was related to the progression of the disease in our series of 119 children with ALL. The group of patients ($n = 28$) with a mean AgNOR value greater than 3 $\mu m^2$ was characterised by a higher number of deaths, more frequent incidence of relapse and shorter time interval to relapse than the group of patients ($n = 91$) with AgNOR area value lower than 3 $\mu m^2$. All these differences were statistically highly significant.

Interphase AgNORs are those nucleolar components in which ribosomal genes are located (Hernandez-Verdun, 1983; Goessens, 1984). Their silver stainability is due to the presence of a specific group of acidic proteins which are necessary for ribosomal RNA synthesis (Howell, 1982). The quantity of interphase Ag ORs greatly increases in the cell stimulated to proliferate. The maximum AgNOR value is reached during the S-phase (Pession *et al.*, 1991). In cancer tissues it has been demonstrated that the quantitative distribution of AgNORs is related to the values obtained using other well-established parameters of cell kinetics such as Ki67 LI (labelling index), bromodeoxyuridine labelling index (BrdU LI) and percentage of S-phase cells determined by flow cytometry (Derenzini & Trerè, 1991). Numerous studies carried out on human tumour cells cultured *in vitro* have shown that the number of AgNORs is strictly and directly related to cell doubling time (Derenzini *et al.*, 1989, 1990; Trerè *et al.*, 1989; Hara *et al.*, 1991; Ofner *et al.*, 1992). Indeed, ribosomal biogenesis necessary for cell duplication is restricted to a shorter period in rapidly dividing cells than in slowing dividing cells with a consequently greater expression of AgNORs in the faster proliferating cells. Interphase AgNOR quantification therefore represents a unique tool to evaluate the rapidity of cell proliferation in routinely processed cytohistological samples. According to the relationship between AgNOR quantity and doubling time of cells cultured *in vitro* (Trerè *et al.*, 1989), the AgNOR values of marrow leukaemic lymphoblasts observed in the present study were consistent with a long doubling time of these cells. The mean AgNOR value of the 119 patients with ALL (2.43 $\mu m^2$) would in fact correspond to a doubling time greater than 100 h. Comparison of the AgNOR quantitative distribution with the pretreatment prognostic factors which are currently used for defining low- and high-risk patients showed that AgNOR values were positively correlated with age, haemoglobin concentration, WBC count and hepatomegaly. The mean AgNOR quantity was greater in the patients with T-cell surface markers than in the non-T group and in L2 than in L1 blasts. An inverse correlation was found between the entity of bone marrow infiltration by blasts and AgNOR values. The latter finding is not surprising: it might be reasonably suggested that the proliferative activity of cancer cells is probably lowered as the neoplastic tissue totally occupies the marrow space.

Other features of the disease, at the time of diagnosis, which have a statistically significative impact on event-free survival of children were, apart from AgNOR value, the immunophenotype and FAB morphology of blasts, the presence of mediastinal tumour mass and leucocyte count. Multiple regression analysis showed that these four parameters are not independent prognostic factors. Only AgNOR value was found to be significantly correlated with the length of event-free survival. This observation is consistent with the correlation between AgNOR value and the single prognostic factors (Table I and II).

The present findings, obtained from a larger number of patients, were consistent with those reported by Scarffe *et al.* (1980) and Dow *et al.* (1982) on the relationship between the proliferative activity of marrow lymphoblasts and the duration of first remission in childhood ALL. These authors found that patients with >6% S-phase cells, measured by either [$^3$H]thymidine incorporation (Dow *et al.*, 1982) or DNA flow cytometry (Scarffe *et al.*, 1980), had a shorter median length of remission than those <6% S-phase cells. In these studies the cell kinetics parameters represented, together with the WBC count, the most powerful prognostic predictors. In the series of patients considered by us, the WBC count lost its significance in the multiple regression analysis. This might have been due either to different therapeutic regimens reducing the prognostic impact of WBC count or/and to the fact that in our study the cell kinetics parameter evaluated was not exactly the same as that measured by Scarffe *et al.* (1980) and Dow *et al.* (1982). AgNOR value indicates the rapidity of cell proliferation, which is different from the percentage of S-phase cells. Even if these two parameters have been shown to be statistically related (Crocker *et al.*, 1988; Tanaka *et al.*, 1989; Orita *et al.*, 1990; Trerè *et al.*, 1991), they cannot be considered to be superimposable. The duration of S-phase has been demonstrated to be variable in marrow leukaemic lymphoblasts, and its value has not always been related to the cell doubling time (Nakamura *et al.*, 1991).

The present findings have demonstrated that cell proliferation is a reliable prognostic parameter in childhood ALL and indicate the opportunity to routinely add cell kinetics evaluation to the other well-established parameters for pretreatment prognostic definition of ALL. Among the methods used for cell kinetics measurement those methods ought to be preferred which evaluate the rapidity of cell proliferation rather than the number of cycling cells. Indeed, it is the former parameter which indicates more precisely the actual growth rate of the neoplastic mass. The importance of the rapidity of cell leukaemic lymphoblast proliferation for the clinical course of ALL can be related to the fact that: (1) if the therapeutic efficacy is the same, the length of the remission would be determined by the degree of cell proliferation rate and (2) drug resistance may develop more quickly in rapidly than in slowly proliferating cells (Scarffe *et al.*, 1980).

Apart from the AgNOR parameter, which has only recently been introduced into tumour pathology for cell kinetics evaluation, cell cycle time length can be precisely measured by DNA flow cytometry (Dolbeare *et al.*, 1983). This procedure, however, necessitates the *in vivo* infusion of BrdU and the exclusive utilisation of one whole-bone marrow aspirate. AgNOR quantification is carried out on a smeared preparation using only a small portion of the biopsy material employed for routine characterisation of the leukaemic marrow infiltrate.

This work was supported by grants from Ministero della Università e della Ricerca Scientifica e Tecnologica (MURST) 40% and 60%, Pallotti's Legacy for Cancer Research and Regione Emilia-Romagna (DGR 4243/1991).

### References

BASSO, G., PUTTI, M.C., CANTU RAJNOLDI, A., SAITTA, M., SANTONASTASI, T., SANTORO, N., LIPPI, A., CORMELLI, A., FULCI, L. & FAURO, C. (1992). The immunophenotype in infant acute lymphoblastic leukaemia: correlation with clinical outcome. An italian multicentre study (AIEOP). *Br. J. Haematol.*, **81**, 184–191.

CHAMPLIN, R. & GALE, P.G. (1989). Acute lymphoblastic leukemia: recent advances in biology and therapy. *Blood*, **73**, 2051–2066.

COX, D.R. (1972). Regression models and life tables. *J.R. Stat. Soc. B.*, **34**, 187–220.

CROCKER, J. (1990). Nucleolar organizer regions. *Curr. Top. Pathol.*, **82**, 91–149.

CROCKER, J., MACARTNEY, J.C. & SMITH, P.J. (1988). Correlation between DNA flow cytometric and nucleolar organizer regions in non-Hodgkin's lymphoma. *J. Pathol.*, **154**, 151–156.

DERENZINI, M. & PLOTON, D. (1991). Interphase nucleolar organizer regions in cancer cells. *Int. Rev. Exp. Pathol.*, **32**, 150–192.

DERENZINI, M. & TRERÈ, D. (1991). Importance of interphase nucleolar organizer regions in tumour pathology. *Virchows Arch. B.*, **61**, 1–8.

DERENZINI, M., PESSION, A., FARABEGOLI, F., TRERÈ, D., BADIALI, M. & DEHAN, P. (1989). Relationship between interphasic nucleolar organizer regions and growth rate in two neuroblastoma cell lines. *Am. J. Pathol.*, **134**, 925–932.

DERENZINI, M., PESSION, A. & TRERÈ, D. (1990). The quantity of nucleolar silver-stained proteins is related to proliferating activity in cancer cells. *Lab. Invest.*, **63**, 137–140.

DOLBEARE, F., GRATZNER, H.G., PALLAVICINI, M.G. & GRAY, J.W. (1983). Flow cytometric measurement of total DNA content and incorporated bromodeoxyuridine. *Proc. Natl Acad. Sci. USA*, **80**, 5573–5577.

DOW, L.W., CHANG, L.J.A., TSIATIS, A.A., MELVIN, S.L. & BOWMAN, W.P. (1982). Relationship of pretreatment lymphoblast proliferative activity and prognosis in 97 children with acute lymphoblastic leukemia. *Blood*, **59**, 1197–1202.

GOESSENS, G. (1984). Nucleolar structure. *Int. Rev. Cytol.*, **87**, 107–158.

HARA, A., NIIKAWA, S., HIRAYAMA, H., SAKAI, N., YAMADA, H., OHNO, T., TANAKA, T. & MORI, H. (1991). Correlation between nucleolar organizer region score and bromodeoxyuridine labeling index in C6 glioma cell line. *J. Neurooncol.*, **11**, 149–155.

HERNANDEZ-VERDUN, D. (1983). The nucleolar organizer regions. *Biol. Cell.*, **49**, 191–202.

HOELZER, D., THIEL, E., LÖFFLER, H., BUCHNER, T., GANSEN, A., HEIL, G., KURRLE, E., HEIMPAL, M., KOCK, P. & LIPP, T. (1988). Prognostic factors in a multicenter study for treatment of acute lymphoblastic leukemia in adults. *Blood*, **71**, 123–131.

HOWELL, W.M. (1982). Selective staining of Nucleolus Organizer Regions (NORs). In *The Cell Nucleus*, Busch, H. & Rothblum, L. (eds) pp. 89–142. Academic Press: New York.

MURPHY, S.B., AUR, R., SIMONE, J., GEORGE, S. & MAUER, A.M. (1977). Pretreatment cytokinetic studies in 94 children with acute leukemia. Relationship to other variables at diagnosis and to outcome of standard treatment. *Blood*, **49**, 683–691.

NAKAMURA, S., TAKEDA, Y., KANNO, M., YOSHIDA, T., OBITAHE, S., KABAYASHI, K., OKABE, Y. & MATSUDA, T. (1991). Application of bromodeoxyuridine (BrdU) and anti-BrdU monoclonal antibody for the in vivo analysis of proliferative characteristics of human leukemic cells in bone marrows. *Oncology*, **48**, 285–289.

OFNER, D., HITTMAIR, A., MARTH, C., ÖFNER, C., TOTSCH, M., DAXENBICHLER, G., MIKUZ, G., MARGREITER, R. & SCHMID, K.W. (1992). Relationship between quantity of silver stained nucleolar organizer region associated proteins (Ag-NORs) and population doubling time in ten breast cancer cell lines. *Pathol. Res. Pract.*, **188**, 742–746.

ORITA, T., KAJIWARA, K., NISHIZAKI, T., IKEDA, N., KAMIRYO, T. & AOKI, H. (1990). Nucleolar organizer regions in meningioma. *Neurosurgery*, **26**, 43–46.

PESSION, A., FARABEGOLI, F., TRERÈ, D., NOVELLO, F., MONTANARO, L., SPERTI, S., RAMBELLI, F. & DERENZINI, M. (1991). The Ag-NOR proteins and transcription and duplication of ribosomal genes in mammalian cell nucleoli. *Chromosoma*, **100**, 242–250.

PLOTON, D., MENAGER, M., JEANNESSON, P., HIMBER, G., PIGEON, F. & ADNET, J.J. (1986). Improvement in the staining and in the visualization of the argyrophilic proteins of the nucleolar organizer region at the optical level. *Histochem. J.*, **18**, 5–14.

ROSSI, M.R., VALSECCHI, A.M. & TESTI, C. (1991). Preliminary report on AIEOP protocol 88 for childhood ALL. *Haematologica*, **76**, 175–184.

SCARFFE, J.H., HANN, I.M., EVANS, D.I.K., MORRIS JONES, P., PALMER, M.K., LILLEYMAN, J.S. & CROWTHER, D. (1980). Relationship between the pretreatment proliferative activity of marrow blast cells and prognosis of acute lymphoblastic leukemia of childhood. *Br. J. Cancer*, **41**, 764–771.

TANAKA, T., TACHEUCHI, T., NISIKAWA, A., TAKAMI, T. & MORI, H. (1989). Nucleolar organizer regions in hepatocarcinogenesis induced by N-2-Fluorenylacetamide in rats: comparison with bromodeoxyuridine immunoistochemistry. *Jpn J. Cancer. Res.*, **80**, 1047–1051.

TRERÈ, D., PESSION, A. & DERENZINI, M. (1989). The silver-stained proteins of interphasic nucleolar organizer regions as a parameter of cell duplication rate. *Exp. Cell. Res.*, **184**, 131–137.

TRERÈ, D., FARABEGOLI, F., CANCELLIERI, A., CECCARELLI, C., EUSEBI, V. & DERENZINI, M. (1991). AgNOR area in interphase nuclei of human tumours correlates with the proliferative activity evaluated by bromodeoxyuridine labeling and Ki 67 immunostaining. *J. Pathol.*, **165**, 53–59.

VAN DER DOES VAN DER BERG, A., BARTRAM, C.R. & BASSO, G. (1992). Minimal requirements for the diagnosis, classification, and evaluation of the treatment of childhood acute lymphoblastic leukemia (ALL) in the 'BMF Family' Cooperative Group. *Med. Pediatr. Oncol.*, **20**, 497–505.

VECCHI, V., PESSION, A. & ROSATI, D. (1990). Preliminary treatment results of '87-AIEOP protocols for childhood acute lymphoblastic leukemia. *Med. Pediatr. Oncol.*, **18**, 403–411.

WOOLSON, R.F. (1987). *Statistical Method for the Analysis of Biomedical Data*. John Wiley: New York.